# Characterizing the lack of diversity in musculoskeletal urgent care website content

**DOI:** 10.1186/s12913-023-09270-5

**Published:** 2023-03-28

**Authors:** Chloe C. Dlott, Tanner Metcalfe, Anchal Bahel, Sanjana Jain, Claire A. Donnelley, Jehanzeb Kayani, Daniel H. Wiznia

**Affiliations:** Department of Orthopaedics & Rehabilitation, Yale University School of Medicine, 800 Howard Avenue, New Haven, CT 06519 USA

**Keywords:** Musculoskeletal urgent care centers, Website content, Racial diversity, Gender diversity, Body type diversity

## Abstract

**Background:**

Musculoskeletal urgent care centers (MUCCs) are becoming an alternative to emergency departments for non-emergent orthopedic injuries as they can provide direct access to orthopedic specialty care. However, they tend to be located in more affluent geographies and are less likely to accept Medicaid insurance than general urgent care centers. MUCCs utilize websites to drive patients to their centers, and the content may influence patients’ consumer behaviors and perceptions of the quality and accessibility of the MUCCs. Given that some MUCCs target insured patient populations, we evaluated the racial, gender, and body type diversity of website content for MUCCs.

**Methods:**

Our group conducted an online search to create a list of MUCCs in the United States. For each MUCC, we analyzed the content featured prominently on the website (above the fold). For each website, we analyzed the race, gender, and body type of the featured model(s). MUCCs were classified according to their affiliation (i.e. academic versus private) and region (i.e. Northeast versus South). We performed chi-squared and univariate logistic regression to investigate trends in MUCC website content.

**Results:**

We found that 14% (32/235) of website graphics featured individuals from multiple racial groups, 57% (135/235) of graphics featured women, and 2% (5/235) of graphics featured overweight or obese individuals. Multiracial presence in website graphics was associated with the presence of women on the websites and Medicaid acceptance.

**Conclusion:**

MUCC website content has the potential to impact patients’ perceptions of medical providers and the medical care they receive. Most MUCC websites lack diversity based on race and body type. The lack of diversity in website content at MUCCs may introduce further disparities in access to orthopedic care.

## Background

Many orthopedic practices utilize websites to provide information about their practices to the public. It has been demonstrated that potential patients who view website content for orthopedic surgeons are more likely to perceive surgeons as competent and more able to provide quality care than surgeons without practice websites [1].

Research has shown that racial minorities respond more favorably to websites that feature similar racial minority models [2–4]. This emphasizes the importance of featuring a diverse selection of models when describing health services in order to cater to all patients. If websites are not diverse in delivering their message, this may contribute to health disparities faced by racial, gender, and weight-based minorities [5–8].

Musculoskeletal urgent care centers (MUCCs) have risen in popularity over the past ten years, in part due to increased wait times for patients at emergency departments [9]. MUCCs are specialized acute care centers focusing on ambulatory orthopedic injuries such as fractures and sprains and offer services such as triage, work up, and imaging [10]. These centers are marketed to patients as an alternative to the emergency department (ED) because they offer fast-track referrals to orthopedic specialists and reduced costs compared to ED visits [11]. The reduced costs and shorter wait times make MUCCs an attractive option to patients and could potentially result in improved patient satisfaction scores [12]. However, MUCCs are known to be preferentially located in affluent areas and can have restrictive policies toward patients with Medicaid, potentially contributing to health disparities [13, 14].

MUCCs utilize websites to drive patients to their centers, and the content may influence patients’ consumer behaviors and perceptions of the quality and accessibility of the MUCCs. Given that some MUCCs target insured patient populations, we evaluated the racial, gender, and body type diversity of website content for MUCCs.

## Methods

### Study design and setting

To investigate the diversity in website content for MUCCs, we performed a comprehensive search for all MUCCs in the United States. In June 2021, our study population was determined and included all MUCCs in the United States located using Google Maps (Mountain View, CA, USA). We used the phrases “XX musculoskeletal urgent care”, “XX orthopedic urgent care”, and “XX MSK urgent care”, where XX was replaced with the two-letter state postal abbreviation. All search results were investigated on Google Maps to determine if they met the classification for a MUCC. Once a MUCC was located on Google Maps, Google (Mountain View, CA) was used to find the MUCC website. In addition, the list of MUCCs we developed was compared to a prior list of MUCCs created by Yousman et al. [14] to ensure all possible MUCCs were included.

### Participants/study subjects

The classification of a MUCC was a clinic that had same-day appointments or walk-in appointments. Orthopedic surgical centers that offered to schedule appointments the next day or the day after were excluded prior to collecting website graphic data. We excluded general urgent care centers and orthopedic clinic offices prior to collecting website graphic data. We found a total of 619 MUCCs in 49 states, excluding Delaware which had no open MUCCs. To collect information from MUCC websites, we examined content on the webpage that did not require scrolling (above the fold). Given that prior research has shown that website users spend more time looking at information above the fold [15, 16], we elected to only include website content that was visible without scrolling on the main page of the website. All website pages were viewed on a 13” laptop screen to standardize the size of the website page. We did not click on additional website tabs to look for other graphics. Videos were excluded. No information was collected on website graphics that did not feature a model or only included body parts such as a hand. If there was not a prominent website graphic, the MUCC was omitted from analysis. A total of 235 prominent website graphics were discovered. As some MUCC websites had multiple locations listed and patients would be able to access the MUCC website through different location specific search terms, we included all MUCC locations in our analyses. Any MUCCs for which we were unable to collect data were excluded from the analysis.

### Variables, outcome measures, data sources, and bias

We collected the following yes/no variables for each prominently featured website graphic (above the fold and visible without scrolling) that included human figures (either full models or partial models with identifiable characteristics) on the website to portray a patient (model)(s): only white model(s), multiracial presence, presence of women, presence of obese/overweight model, white feature model, and female feature model. We selected race/ethnicity, gender, and body habitus as our primary variables given that prior research in orthopedic surgery has demonstrated that patients from racial or ethnic minority backgrounds, women, and obese patients may have difficulty accessing orthopedic care and may have worse outcomes following orthopedic surgery [17–19]. To minimize bias, two authors reviewed each graphic to collect the above variables and a third author was included if any disagreement regarding the variables arose, which occurred infrequently. A study by Tirrell et al. utilized an author as a reviewer to assess race and gender of models included in social media posts by various plastic surgery groups [20]. Rankin et al. used a similar methodology including the use of two author reviewers for direct-to-consumer advertising in total joint arthroplasty [21]. Prior research has demonstrated that APIs used to classify race/ethnicity and gender of graphics can have low accuracy which is why we utilized multiple reviewers instead of an API [22, 23]. Multiracial presence was defined as when a website graphic portrayed individuals from multiple racial groups (including White, Hispanic, Black, and Asian) at the same level of perspective. A graphic would not qualify for multiracial presence if a white model was the focus with models with different races in the background; all individuals had to be featured equally. A feature model was defined as the model that was most prominent in the graphic either by being the largest graphic or being in the foreground of the graphic. If a graphic included models from multiple racial groups with a white model in the foreground, then this graphic was classified as having a white feature model. Rankin et al. used a similar classification scheme with non-white focused advertisements which are equivalent to multiracial graphics in this study and white-focused advertisements which are equivalent to white feature model graphics in this study [21]. We chose to use this classification scheme as we felt that graphics with white models in the foreground with multiple racial groups in the background does not portray true equity in representation. As in Rankin et al. [21], the presence of overweight/obese models was determined as when models had an estimated body mass index of greater than 30. While this is a more subjective measure, each graphic was reviewed by two of the authors and confirmed by a physician.

Individual MUCCs were classified according to their affiliation as either nonaffiliated (without a connection to a hospital or practice), extension (a MUCC associated with a private practice or nonacademic hospital), or academic (associated with a teaching hospital). They were also classified according to their geographic region (Northeast, South, Midwest, West) according to the U.S. Census definitions of region [24]. Cash payment prices (the required payment for an appointment for a patient without insurance) for individual MUCCs were collected in a previous study by Yousman et al. [14].

### Primary and secondary study outcomes

Our primary study goal was to characterize the racial, gender, and body type diversity present in website content for MUCCs across the United States. To achieve this, we collected website graphic information as explained above and identified the race, gender, and body type of the model(s) portrayed in each graphic.

Our secondary study goal was to analyze what factors increased the likelihood of diverse website content such as MUCC affiliation, MUCC region, and cash payment cost. We achieved this by performing analysis to investigate which factors were most strongly associated with increased website content diversity.

### Statistical analysis

Chi-squared and univariate logistic regression analyses were performed to analyze trends in MUCC website content and to investigate factors that were associated with increased diversity in website graphics. All statistical analysis was performed using Stata Version 16.1 (StataCorp, College Station, TX).

## Results

### Overview

Overall, 14% (32/235) of MUCCs had graphics on their websites that included individuals from multiple racial groups at equal levels of perspective, 80% (188/235) of graphics featured only white models, 57% (135/235) of graphics included women, 2% (5/235) included obese or overweight models, 86% (203/235) of graphics had a white feature model, and 34% (81/235) of graphics had a female feature model (Table 1).

### Differences in website graphics by region

Website graphics with a white feature model differed by region (p < 0.01) with the South having the highest proportion of website graphics with white feature models and the Northeast having the lowest proportion. Website graphics with a female feature model differed by region (p < 0.01) with the Northeast having the highest proportion of website graphics that had a female model as the most predominant figure in the graphic and the South having the lowest proportion. There were no differences in the proportion of website graphics that included body type diversity between geographic regions in the United States (p > 0.05) (Table 1).

**Table 1:** Website Graphic Content Patterns of Musculoskeletal Urgent Care Centers

		Only White Model(s) n (%)	Multiracial Presence n (%)	Presence of Women n (%)	Presence of Obese/Overweight Model(s) n (%)	White Feature Model n (%)	Female Feature Model n (%)
Overall	N = 235	188 (80%)	32 (14%)	135 (57%)	5 (2%)	203 (86%)	81 (34%)
Region	Northeast (n = 67)	48 (72%)	13 (19%)	43 (64%)	1 (1%)	50 (75%)	34 (51%)
	Midwest (n = 48)	37 (77%)	8 (17%)	32 (67%)	1 (2%)	43 (90%)	16 (33%)
	South (n = 88)	78 (89%)	7 (8%)	42 (48%)	2 (2%)	82 (93%)	20 (23%)
	West (n = 32)	25 (78%)	4 (13%)	18 (56%)	1 (3%)	28 (88%)	11 (34%)
Region P-value		0.06	0.19	0.10	0.96	**p < 0.01**	**p < 0.01**
Affiliation	Private Practice (n = 206)	168 (82%)	24 (12%)	113 (55%)	5 (2%)	183 (89%)	66 (32%)
	Academic Hospital (n = 16)	11 (69%)	5 (31%)	12 (75%)	0 (0%)	11 (69%)	9 (56%)
	No Affiliation (n = 13)	9 (69%)	3 (23%)	10 (77%)	0 (0%)	9 (69%)	6 (46%)
Medicaid Acceptance	Yes (n = 158)	120 (76%)	28 (18%)	95 (60%)	4 (3%)	133 (84%)	53 (34%)
	No (n = 77)	68 (88%)	4 (5%)	40 (52%)	1 (1%)	70 (91%)	28 (36%)
Average Cash Payment Price	n = 205* $ ± SD	$251 ± 95	$233 ± 85	$245 ± 98	$282 ± 103	$252 ± 93	$245 ± 98

SD: standard deviation

*Cash payment price was not available for 30 musculoskeletal urgent care centers

### Differences in graphics by Medicaid acceptance and cash payment price

For graphics at MUCCs that accepted Medicaid, 76% (120/158) included only white models, 18% (28/158) had multiracial presence, 60% (95/158) included women, 3% (4/158) included obese/overweight models, 84% (133/158) included white feature models, and 34% (53/158) included female feature models. For graphics at MUCCs that did not accept Medicaid, 88% (68/77) included only white models, 5% (4/77) had multiracial presence, 52% (40/77) included women, 1% (1/77) included obese/overweight models, 91% (70/77) included white feature models, and 36% (28/77) had female feature models. The average cash payment price at the 205 MUCCs for which we had data was $251 (standard deviation (SD): 95) for MUCCs with graphics that included only white models, $233 (SD: 85) with graphics that had multiracial presence, $245 (SD: 98) with graphics that included women, $282 (SD: 103) with graphics that included obese/overweight models, $252 (SD: 93) for graphics with white feature models, and $245 (SD: 98) for graphics with female feature models (Table 1).

### Factors associated with multiracial presence in website graphics

MUCCs that accepted Medicaid were more likely to have a multiracial presence in their website graphics (OR = 1.11, 95% CI: 1.00–1.22, p = 0.04). Additionally, the presence of women in website graphics was associated with an increased likelihood of multiracial presence (OR = 1.18, 95% CI: 1.08–1.29, p < 0.001). Region, MUCC affiliation, and cash payment price were not associated with multiracial presence in website content (Table 2).

**Table 2:** Factors Associated with Multiracial Presence in Website Graphics (n = 205)*

Factor	Odds ratio	p-value	95% confidence interval
Region	0.97	0.28	[0.93, 1.02]
Affiliation	1.02	0.73	[0.90, 1.16]
Cash Payment Price*	1.00	0.41	[0.99, 1.00]
Medicaid Acceptance	1.11	**0.04**	[1.00, 1.22]
Presence of Women	1.18	**p < 0.001**	[1.08, 1.29]

*Cash payment price only available for 205 musculoskeletal urgent care centers

### Factors associated with women in website graphics

Websites that had a multiracial presence in their graphics were also more likely to feature women (OR 1.44, 95% CI: 1.18–1.76, p < 0.001). Region, MUCC affiliation, cash payment price, and MUCC Medicaid acceptance were not associated with the presence of women in website content (Table 3).

**Table 3:** Factors Associated with Presence of Women in Website Graphics (n = 205)*

Factor	Odds ratio	p-value	95% confidence interval
Region	1.01	0.87	[0.94, 1.08]
Affiliation	0.96	0.68	[0.79, 1.16]
Cash Payment Price*	1.00	0.43	[1.00, 1.00]
Medicaid Acceptance	1.02	0.81	[0.88, 1.18]
Multiracial Presence	1.44	**p < 0.001**	[1.18, 1.76]

*Cash payment price only available for 205 musculoskeletal urgent care centers

## Discussion

MUCC website content has the potential to impact patients’ perceptions of medical providers and the medical care they receive. Given that patients are more likely to respond to marketing content that reflects their personal demographic background, the lack of diversity in website graphics at MUCCs may introduce further disparities in access to orthopedic care [1–4, 25]. Our group sought to further explore and add to the minimal research that has been conducted on MUCC website content related to racial, gender, and body type diversity. Our study found limited diversity in website graphics for MUCCs with only 14% of graphics featuring multiracial presence, 80% of graphics featuring only white models, 57% of graphics featuring women, and only 2% of graphics featuring an overweight/obese individual. MUCCs that accepted Medicaid were more likely to have website graphics with multiracial presence which may indicate that they are attempting to provide service to a more diverse population. Cash payment price was not significantly associated with the presence of women in website graphics or multiracial presence in website graphics. More than half of MUCC websites did not include graphics and as such were excluded from our analysis. This may indicate that these MUCCs either had insufficient funds or did not prioritize website content that included models.

A lack of racial diversity in medical marketing has been demonstrated in prior studies [20, 21]. Direct-to-consumer marketing is most effective when it reflects a patient’s own demographic background [26]. In our study, racial diversity was examined by considering both the inclusion of multiracial presence in website graphics and the inclusion of website graphics that only included white models. It is especially concerning that MUCCs in the South are less likely to include individuals from racially diverse backgrounds in their website graphics given the high racial diversity present in this region compared to the overall population [27]. Patients may be less likely to seek care at MUCCs that they feel are not treating patients with similar characteristics. Minority patients face many barriers to orthopedic care including decreased access, provider bias, and insurance status [6, 13, 14, 28, 29]. Our data supports that MUCCs do not include content marketed to minority patients as 80% of website graphics included only white models while only 14% featured a multiracial presence. Similar findings were reported by Rankin et al. [21], who found that non-white-focused direct-to-consumer advertisements were utilized by total joint replacement medical device companies approximately 14% of the time and Tirrell et al. [20] who found that overall approximately 88% of patient images displayed white skin tones and 12% displayed nonwhite skin tones.

Our study found that 57% of website graphics featured women. This is encouraging as it reflects that women are an integral part of orthopedic care. Women are more likely than men to present to the ED and are more likely to experience musculoskeletal related morbidities and pain [9, 30, 31]. Women have the potential to benefit from the specialized orthopedic care provided at MUCCs yet may not seek care for orthopedic concerns if they feel that MUCCs do not provide care for women.

Our study found that 2% of website graphics featured an obese/overweight model. This is similar to the findings of Rankin et al. who found that approximately 3% of models included in direct-to-consumer advertisements for total joint arthroplasty were obese [21]. As 40% of the population in the United States is obese, MUCC website graphics do not capture the diversity present in the general population [32]. Prior research has demonstrated that obese/overweight individuals experience stigma related to their weight which may lead to decreased care utilization [33, 34]. MUCCs may be creating further barriers to care utilization for overweight/obese patients by employing marketing practices that exclude models with diverse body types. Focusing on increasing body type diversity in website graphics could be a starting point to increase overall diversity.

MUCCs that accept Medicaid and MUCCs with website graphics that included women were associated with an increased likelihood of multiracial presence. Patients who identify as Black, Hispanic, and Asian are more likely to be insured by Medicaid [35] and perhaps MUCCs that accept Medicaid are trying to encourage patients from racial and ethnic minority backgrounds to seek care at their clinics. Additionally, as the presence of women is associated with an increased likelihood of multiracial presence, it is possible that diversity in one domain, such as gender, promotes diversity in other domains, such as race. As multiracial presence in website graphics is associated with an increased likelihood of the presence of women in website graphics, this reinforces that diversity in one domain encourages diversity in another.

### Limitations

Our study has several limitations. First, as no centralized database for MUCCs exists, our search strategy may have missed some MUCC locations given that Google Maps is limited to registered buildings and is partially crowd sourced. However, we feel that our search was comprehensive and encompassed all MUCCs that could be found on the internet. In addition, we were able to compare our list with a previous list produced by Yousman et al. [14] which provided us with a way to further search for MUCCs. Second, it is possible that we misclassified the perceived race/ethnicity or body type of models featured in the website graphics. We minimized this risk by having multiple authors review the website graphics and come to a consensus when a disagreement arose, which occurred infrequently. Misclassification of perceived race/ethnicity or gender could be more likely if the graphic did not include a full human figure but instead included only a partial human figure. However, a very small number of graphics did not include full human figures and we only included graphics with partial figures if they had identifiable characteristics. We excluded graphics that only had specific body parts such as a hand as the risk of misclassification would be higher. Third, we only analyzed graphics that were above the fold. It is possible that websites included graphics on other parts of the website. However, given that most people focus on content above the fold [15, 16], we felt this was a reasonable criterion for data collection. In addition, all data was collected on 13” laptops to try and standardize the website content that was visible without scrolling.

## Conclusion

The lack of diversity in website content at MUCCs may introduce further disparities in access to orthopedic care. We recommend that MUCCs feature more diverse graphics on their websites with the goal of reducing disparities in orthopedic urgent care for minority patients. Further studies characterizing the impact of MUCC website graphics on patient utilization should be investigated such as patient surveys that directly ask patients which factors, including website graphics, would impact their utilization of MUCCs. In addition, further studies could focus on other aspects of diversity such as disability status or patient age and other possible factors that could impact diversity such as urban/rural location and diversity of providers at each MUCC.

## Data Availability

The datasets used and/or analyzed during the current study are available from the corresponding author on reasonable request.

## Abbreviations

ED: emergency department
MUCC: musculoskeletal urgent care center
SD: standard deviation
